# MELD 3.0 score in prediction of varices and comparison with its previous versions in patients undergoing esophago-gastro-duodenoscopy for variceal screening or band ligation

**DOI:** 10.12669/pjms.41.10.11193

**Published:** 2025-10

**Authors:** Faiza Sadaqat Ali, Bader Faiyaz Zuberi, Tazeen Rasheed, Niaha, Amanullah Abbasi

**Affiliations:** 1 Faiza Sadaqat Ali, FCPS Senior Registrar, Department of Medicine/Gastroenterology, Dow Medical College, Dow University of Health Sciences, Karachi, Pakistan; 2 Bader Faiyaz Zuberi, FCPS Meritorious Professor, OMI Hospital, Saddar Karachi, Pakistan; 3 Tazeen Rasheed, FCPS Associate Professor, Department of Medicine/Gastroenterology, Dow Medical College, Dow University of Health Sciences, Karachi, Pakistan; 4 Niaha, MBBS Postgraduate Trainee, Department of Medicine/Gastroenterology, Dow Medical College, Dow University of Health Sciences, Karachi, Pakistan; 5 Amanullah Abbasi, FCPS Meritorious Professor, Department of Medicine/Gastroenterology, Dow Medical College, Dow University of Health Sciences, Karachi, Pakistan

**Keywords:** Cirrhosis, MELD, MELDNa, MELD 3.0, Varices, Variceal Prediction

## Abstract

**Objective:**

To compare MELD, MELDNa & MELD 3.0 score values with presence of varices in patients undergoing esophago-gastro-duodenoscopy for variceal screening or band ligation. The other objective was to estimate cut-off values of various MELD variants in prediction of varices using Area Under Receiver Operator Curve (AUROC).

**Methodology:**

This cross-sectional study was conducted in Department of Medicine/Gastroenterology at Dow Medical College, Karachi during the period 20th November 2022 to 19th September 2024. All patients of either gender of age between 18 to 65 years undergoing screening endoscopy were included in the study after informed consent. MELD, MELDNa & MELD 3.0 scores were calculated, grades of oesophageal varices were recorded.

**Results:**

Total of 321 patients were included. Highest scores for presence of varices were reported by MELD 3.0 (19.21). No difference was found for variceal presence on gender in MELD 3.0 scores but MELD & MELDNa scores were significantly lower in females. All MELD variants showed significant area under curve on ROC with highest AUC in MELDNa. Regression analysis for MELD variants showed that 88.8% of MELD 3.0 score was influenced by age, gender, bilirubin, sodium, INR, creatinine & albumin. Age, sodium & albumin affected negatively on the score. In both MELD and MELDNa scores albumin levels did not have any significant effect on score.

**Conclusions:**

All MELD variants have good predictability for varices at their different scores with MELDNa showing highest AUC. MELD 3.0 gives more weightage to female gender by allocating them score that are not different from males, while the other two allocate lower scores to females.

## INTRODUCTION

The Model for End-Stage Liver Disease (MELD) is a widely used scoring system to assess the severity of chronic liver disease and predict short-term survival. MELD calculates risk based on serum creatinine, total bilirubin, and INR, and has played a crucial role in prioritizing liver transplant recipients since 2002.^1^,^2^ Over time, however, its predictive accuracy declined, particularly for patients with metabolic-associated steatotic liver disease and alcohol-related cirrhosis, though it remained reliable in post-viral and cholestatic liver diseases.^3^,^4^

To improve predictive capability, MELDNa was developed, incorporating serum sodium into the calculation.^5^ This adaptation enhanced accuracy and is now standard for risk assessment and transplant eligibility. However, MELDNa was found to underestimate mortality risk in women, as creatinine levels often overestimated their renal function, disadvantaging them in organ allocation.^6^

To address this, MELD 3.0 was introduced in 2023, adding female gender and serum albumin to the calculation, including an additional 1.33 points for female patients.^4^,^7^,^8^ This adjustment led to 8.8% of subjects, especially women, being assigned to a higher transplant priority tier and reduced waiting list deaths in simulation studies.^7^ MELD 3.0 also lowered the ceiling for serum creatinine and incorporated interaction terms between creatinine and albumin, as well as between sodium and bilirubin, further improving mortality prediction.^7^

While MELD 3.0’s effectiveness in transplant allocation is validated, its value in predicting variceal bleeding has not yet been extensively studied. This study aimed to compare MELD, MELDNa, and MELD 3.0 scores with the presence and grades of oesophageal varices in both genders, and to determine optimal cut-off values using AUROC. These findings could help physicians predict variceal presence non-invasively and inform better treatment decisions.

## METHODOLOGY

This cross-sectional study was conducted in in-patients and out-patients at Department of Medicine/Gastroenterology at Dow Medical College, Karachi during the period 20^th^ November 2022 till 19^th^ September 2024.

All patients of either gender of age between 18 to 65 years undergoing screening endoscopy or band ligation for first time were included in the study. Patients suffering from severe cardiac, respiratory or psychiatric disease, splenic or portal vein thrombosis, hepatocellular carcinoma, primary biliary cirrhosis, INR ≥1.8, platelets count of <50,000 & history of previous band ligation or sclerotherapy were excluded. Sample Size was calculated using the reported discrimination of MELDNa of 0.862 versus MELD 3.0 of 0.869.^7^ Calculation was done for two proportions using Z-test with un-pooled variance, keeping power at 0.90 and alpha at 0.05, sample size was computed as 253. Sample size computation was done using PASS software.

### Ethical Approval:

It was obtained from Dow University of Health Sciences Ref. # IRB-2720/DUHS/Approval/2022/1112; Dated: 14^th^ November 2022.

### Operational Definitions:

Calculations of MELD, MELDNa & MELD 3.0 scores was done as per standard respective formulas^7^ using online free calculator offered by Stanford University (Stanford, CA, 94305, USA).^9^

### Variceal Staging:

Small Varices: ≤5 mm; Large Varices >5 mm.^10^

### Data Collection Procedure:

Informed written consent was taken from all patients Selected patients underwent a detailed clinical examination and laboratory tests including CBC, PT & INR, urea/creatinine/electrolytes (UCE), liver functions tests (LFT) and serum albumin (Alb). EGD was done using standard protocol with conscious sedation after 6 hours fast. Reporting of staging of varices was done on EGD and MELD, MELDNa & MELD 3.0 were calculated as described in operational definitions.

### Data Analysis Procedure:

Quantitative variables were expressed in mean ±SD while qualitative variables were reported in frequencies and percentages. Χ^2^ test was done to compare frequencies and types of varices with gender, and means of age, bilirubin, sodium, INR, creatinine, albumin, and MELD score variants were compared by gender using Student’s t-test. Means of MELD variants were compared by variceal categories and gender using Student’s t-test. Correlation between MELD variants and variceal categories was done using Pearsons Correlation test. Receiver Operating Characteristic (ROC) curves were plotted and Area Under Curve (AUC) was calculated MELD variants for variceal categories. Cut-off values of MELD variants with sensitivity and 1-specificity (chance of true negative testing positive) were determined for variceal categories detection. Regression analysis was conducted for MELD variants as dependent variable (DV) and age, gender, bilirubin, sodium, INR, creatinine & albumin as Independent Variables (IV), R^2^ values were reported. The heteroscedasticity & normality of IV was checked by plotting histogram, PP plots and by plotting a scatter graph of standardized residuals against standardized predictable. Statistical analysis was achieved using SPSS (Version 27.0; IBM, New York, NY). Significance level was set at ≤.05.

## RESULTS

Three hundred twenty-one patients were inducted into the study. These included 173 (53.9%) males (age 46.94 ±10.79 years) and 148 (46.1%) females (age 48.39 ±17.18 years), no significant difference in age was found between gender t (239.7) = -.88, p = .376. Varices were present in 291 (90.65%) in patients, out of these 160 were males and 131 were females. Frequencies of varices and their categories were compared with gender using χ^2^ test that showed significantly more frequency of varices in males (Table-I). Comparison of age, bilirubin, Sodium, INR, Creatinine, Albumin, MELD, MELDNa, MELD 3.0 scores was done by gender using Student’s t-test. It showed significantly lower score of MELD and MELDNa in females but no significant difference in MELD 3.0 scores (Table-II). MELD scores were compared for presence/absence of varices according to gender.

**Table-I:** Cross-tabulation of varices with gender and χ^2^ test.

		Varices Absent	Varices Present				Grand Total
			Small	Large	Gastric Varix	Sub-Total	
Gender	Male	13 (43.3)	31 (36.0)	120 (62.8)	9 (64.3)	160 (49.8)	173 (53.9)
	Female	17 (56.7)	55 (64.0)	71 (37.2)	5 (35.7)	131 (40.8)	148 (46.1)
Total		30 (100.0)	86 (100.0)	191 (100.0)	14 (100.0)	291 (90.7)	321 (100.0)

χ^2^ = (df = 3, N = 321) = 19.1; p <.001, Significance ≤.05.

**Table-II:** Comparison of quantitative variables by gender using Student’s Test.

	Gender					
	Male		Female		Combined	
	Mean	±SD	Mean	±SD	Mean	±SD
Age (years)	47.12_a_	11.00	48.19_a_	17.04	47.61	14.09
Total Bilirubin (mg/dl)	2.03_a_	1.85	1.91_a_	1.81	1.96	1.83
Sodium (mmol/L)	133.10_a_	5.18	136.66_b_	9.86	134.74	10.32
INR	1.44_a_	.27	1.39_a_	.30	1.42	0.29
Creatinine (mg/dl)	1.40_a_	.95	1.30_a_	1.26	1.35	1.10
Albumin (mg/dl)	2.36_a_	.56	2.44_a_	.68	2.40	0.62
MELD Score	15.90_a_	5.46	13.79_b_	5.95	14.93	5.78
MELDNa Score	18.45_a_	6.42	15.15_b_	6.56	16.93	6.68
MELD 3.0 Score	19.49_a_	6.31	18.89_a_	8.09	19.21	7.18

***Note:*** Values in the same row and sub-table not sharing the same subscript are significantly different at p < .05 in the two-sided test of equality for column means. Cells with no subscript are not included in the test. Tests assume equal variances.

**Fig.1:**
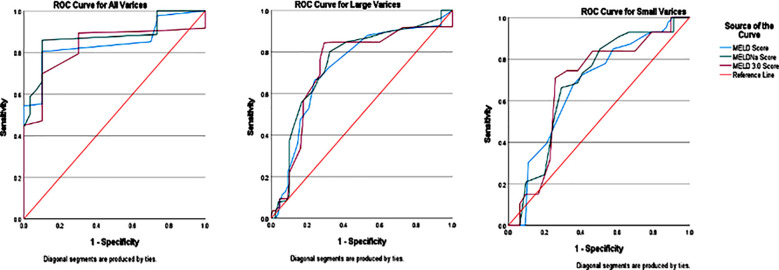
ROC Curve of all varices, large varices and small varices for MELD, MELDNa & MELD 3.0.

Statistically significant lower score was allocated to female gender by MELD & MELDNa, but no significant difference was present between gender in MELD 3.0 (Table-III). Correlation Matrix of MELD, MELDNa, MELD 3.0 scores, Variceal Categories and Variceal presence is given in Table-IV, it shows highly significant positive correlations between all variables. ROC curve was plotted for all categories of varices MELD, MELDNa & MELD 3.0 scores (Fig.1) and all showed significant AUC, but MELDNa showed highest AUC for all categories of varices (Table-V). Cut-off values of MELD, MELDNa & MELD 3.0 with sensitivity & 1-specificity for variceal prediction are given in Table-VI. Regression analysis for MELD variants was conducted to see the quantification of effects of IV (age, gender, bilirubin, sodium, INR, creatinine & albumin). The adjusted R^2^ value for MELD 3.0 was .888 meaning that 88.8% of MELD 3.0 score was influenced by IV. All IV had significant influence on MELD 3.0 scores. Age, sodium & albumin affected negatively on the score. In both MELD and MELDNa scores albumin levels did not have any significant effect on score. Details of regression analysis of all MELD variants is given in Table-VII.

**Table-III:** Comparison of different MELD Scores using Student’s t-test for presence/absence of varices according to gender.

	Varices			
	Absent		Present	
	Male	Female	Male	Female
MELD Score	9.92_a_	9.29_a_	16.38_a_	14.37_b_
MELDNa Score	10.00_a_	9.59_a_	19.13_a_	15.87_b_
MELD 3.0 Score	12.00_a_	13.65_a_	20.09_a_	19.57_a_

***Note:*** Values in the same row and sub-table not sharing the same subscript are significantly different at p < .05 in the two-sided test of equality for column means. Cells with no subscript are not included in the test. Tests assume equal variances.

**Table-IV:** Correlation Matrix of MELD, MELDNa, MELD 3.0 scores, Variceal Categories and Variceal presence.

		MELD Score	MELDNa Score	MELD 3.0 Score	Varices Categories
MELDNa Score	Corr.	.950	--		
	Sig.	<.001**			
MELD 3.0 Score	Corr.	.936	.950	--	
	Sig.	<.001**	<.001**		
Varices Categories	Corr.	.464	.504	.455	--
	Sig.	<.001**	<.001**	<.001**	
Varices (Present/Absent)	Corr.	.298	.345	.281	.710
	Sig.	<.001**	<.001**	<.001**	<.001**

** . Correlation is significant at the 0.01 level (2-tailed).

**Table-V:** Area under Curve for various variceal categories for MELD, MELDNa & MELD3.0 Scores.

Test Result Variables	All Varices			Large Varices			Small Varices		
	AUC	SE^a^	Sig.^b^	AUC	SE^a^	Sig.^b^	AUC	SE^a^	Sig.^b^
MELD Score	.845	.027	<.001	.725	.030	<.001	.673	.032	<.001
MELDNa Score	.877	.026	<.001	.747	.029	<.001	.684	.032	<.001
MELD 3.0 Score	.827	.030	<.001	.732	.031	<.001	.679	.033	<.001

AUC = Area Under Curve, SE = Standard Error. a. Under the nonparametric assumption. b. Null hypothesis: true area = 0.5.

**Table-VI:** Cut-off values with Sensitivity & 1-Specificity for prediction of MELD, MELDNa & MELD 3.0 Scores for various categories of varices.

		≥ Positive	Sensitivity	1-Specificity
All varices	MELD	7.50	.976	.733
	MELDNa	8.50	.976	.733
	MELD 3.0	11.50	.904	.733
Large varices	MELD	7.50	.963	.938
	MELDNa	8.50	.963	.938
	MELD 3.0	10.50	.921	.931
Small varices	MELD	9.50	.698	.889
	MELDNa	9.50	.791	.902
	MELD 3.0	10.50	.895	.936

**Table-VII:** Regression analysis with MELD Variants as dependent variables and age, gender, bilirubin, sodium, INR, creatinine & albumin as independent variables.

Model		Unstandardized Coefficients		Standardized Coefficients	t	Sig.	Adjusted R^2^
		B	SE	Beta			
MELD	(Constant)	18.273	2.102		8.695	<.001	.903
	Bilirubin*	.794	.072	.251	10.976	<.001	
	INR*	7.788	.451	.387	17.258	<.001	
	Creatinine*	2.758	.111	.526	24.865	<.001	
	Age	-.018	.008	-.045	-2.447	.015	
	Gender	-.838	.206	-.072	-4.066	<.001	
	Sodium	-.128	.013	-.229	-9.611	<.001	
	Albumin	-.135	.182	-.014	-.743	.458	
MELDNa	(Constant)	44.589	2.544		17.524	<.001	.893
	Bilirubin*	.600	.088	.164	6.853	<.001	
	INR*	7.460	.546	.321	13.654	<.001	
	Creatinine*	2.454	.134	.405	18.273	<.001	
	Sodium *	-.292	.016	-.451	-18.091	<.001	
	Age	-.039	.009	-.082	-4.285	<.001	
	Gender	-1.528	.249	-.114	-6.126	<.001	
	Albumin	.276	.220	.026	1.253	.211	
MELD 3.0	(Constant)	43.832	2.799		15.659	<.001	.888
	Gender*	1.377	.274	.096	5.019	<.001	
	Bilirubin*	.624	.096	.159	6.470	<.001	
	Sodium*	-.303	.018	-.436	-17.090	<.001	
	INR*	8.976	.601	.359	14.935	<.001	
	Creatinine*	2.585	.148	.397	17.496	<.001	
	Albumin*	-.531	.242	-.046	-2.190	.029	
	Age	-.041	.010	-.080	-4.096	<.001	

Significance ≤.05, SE = Standard Error,

* . Independent variables that are part of score.

## DISCUSSION

Our study showed that MELD 3.0 is gender neutral and gives similar scores to both genders while MELD & MELDNa placed females at disadvantage. The study rigorously compared three prominent MELD score variants (MELD, MELDNa, MELD 3.0) in predicting variceal presence and severity, offering a nuanced evaluation seldom undertaken in prior research. Kim et al. introduced MELD 3.0 with the aim to enhance waitlist mortality modelling and improve allocation for liver transplantation,^7^ notably by removing eGFR as a variable and incorporating female gender.^11^ Many prognostic models, such as MELD, MELDNa, and Child-Turcotte-Pugh (CTP), rely on clinical and laboratory parameters to risk-stratify patients during bleeding presentations. This study evaluates three MELD variants in predicting varices, using endoscopic findings as the standard.

We observed that rising MELD, MELDNa, and MELD 3.0 scores correlated positively with increased severity of varices. Importantly, MELD and MELDNa assigned lower scores to females, while MELD 3.0 resolved this bias and scored similarly across genders. This addresses the known issue of under-allocation for females in earlier models, an improvement also reflected in the GEMA-Na score.^12^ Our objective was to determine the predictive power of MELD 3.0 for varices and compare it to previous versions. Prior research by Fong TV, et al. found a MELD score of 13 predictive for varices;^13^ our study identified a MELD 3.0 score of 11.50 and a MELD score of 7.50 for varices prediction, with strong sensitivity and specificity. Differences with earlier studies may be due to varying demographics and prevalence of alcohol-related cirrhosis.^14^

Luca A et al. suggested that sodium and age improve mortality prediction by boosting MELD scores.^15^ Our regression analysis showed that age and sodium negatively influenced all MELD variants, although age is not included as a variable in current MELD scores. This finding highlights the potential value in considering age in future versions. MELD 3.0 had the lowest reported waitlist death rate (5.8%) compared to MELDNa (6.7%) and MELD (12.0%).^4^

Although all three models demonstrated significant AUCs for varices prediction, MELDNa had the highest across all categories. Wang J et al.^16^ reported that MELDNa is clinically useful in predicting rebleeding and mortality in cirrhotic patients’ post-endoscopic therapy, supporting our findings. Hepatic venous pressure gradient (HVPG) has also shown strong positive correlation with MELD and variceal grades.^17^

Despite advances, MELD scores do not account for factors such as malnutrition, sarcopenia, and frailty—areas where the Liver Frailty Index (LFI) offers additional prognostic value.^18^ Technological developments, including AI-based data-driven algorithms and new indicators like GRAIL, may further refine outcome predictions.^19^-^22^ By correlating MELD variants with variceal grades and demonstrating their predictive power through ROC curves and sensitivity/specificity analysis, the study provides valuable information for risk stratification in cirrhotic patients.

### Limitations:

This being a single center study, results could not be generalized to the general population. Due to its a cross-sectional design outcome of the patients could not be validated prospectively. Comparisons with frailty and sarcopenic status of patients was not assessed.

## CONCLUSIONS

The study compares various MELD variants and finds that MELD 3.0 gives higher score to females as compared to its previous versions in our settings. Significant positive correlation existed between all MELD variants with grades of varices and among themselves. Significant AUC were present with all variants of MELD with best values present with MELDNa.

### Authors’ Contribution:

**FSA:** Conception and design of study.

**BFZ:** Final Approval, statistical analysis.

**TR**: Manuscript writing.

**N:** Data collection, initial draft writing.

**AA:** Manuscript editing, data collection.

All authors have read and approved the final version. They are also responsible for accuracy and integrity of work.

## References

[1] Malinchoc M, Kamath PS, Gordon FD, Peine CJ, Rank J, Ter Borg PC (2000). A Model to Predict Poor Survival in Patients Undergoing Transjugular Intrahepatic Portosystemic Shunts. Hepatology.

[2] Sarwar S, Khan AA, Tarique S (2008). Comparison of Meld, Child Pugh Score and Rockall Score for Predicting Rebleeding and in-Hospital Mortality in Patients of Variceal Bleeding. J Coll Physicians Surg Pak.

[3] Godfrey EL, Malik TH, Lai JC, Mindikoglu AL, Galvan NTN, Cotton RT (2019). The Decreasing Predictive Power of Meld in an Era of Changing Etiology of Liver Disease. Am J Transplant.

[4] Zaver HB, Rajpal N, Shah NL, Argo CK (2024). Meld and Meld 3.0:What It Means for Your Practice. Am J Gastroenterol.

[5] Tejedor M, Selzner N, Berenguer M (2022). Are Meld and Meldna Still Reliable Tools to Predict Mortality on the Liver Transplant Waiting List?. Transplantation.

[6] Locke JE, Shelton BA, Olthoff KM, Pomfret EA, Forde KA, Sawinski D (2020). Quantifying Sex-Based Disparities in Liver Allocation. JAMA Surg.

[7] Kim WR, Mannalithara A, Heimbach JK, Kamath PS, Asrani SK, Biggins SW (2021). Meld 3.0:The Model for End-Stage Liver Disease Updated for the Modern Era. Gastroenterology.

[8] Ballotin VR, Bigarella LG, Riva F, Onzi G, Balbinot RA, Balbinot SS (2020). Primary Sclerosing Cholangitis and Autoimmune Hepatitis Overlap Syndrome Associated with Inflammatory Bowel Disease:A Case Report and Systematic Review. World J Clin Cases.

[9] Medicine S (2021). Medical Calculators Stanford.

[10] Koh C, Zhao X, Samala N, Sakiani S, Liang TJ, Talwalkar JA (2013). Aasld Clinical Practice Guidelines:A Critical Review of Scientific Evidence and Evolving Recommendations. Hepatology.

[11] Mazumder NR, Fontana RJ (2024). Meld 3.0 in Advanced Chronic Liver Disease. Annu Rev Med.

[12] Rodriguez-Peralvarez ML, De la Rosa G, Gomez-Orellana AM, Aguilera MV, Pascual Vicente T, Pereira S (2024). Gema-Na and Meld 3.0 Severity Scores to Address Sex Disparities for Accessing Liver Transplantation:A Nationwide Retrospective Cohort Study. EClinicalMedicine.

[13] Fong TV, Hung FC, Chiu KW, Chiu YC, Wu KL, Kuo CH (2008). Model for End-Stage Liver Disease (Meld) Score for Predicting Late Esophageal Varices Rebleeding in Cirrhotic Patients. Hepatogastroenterology.

[14] Sheth M, Riggs M, Patel T (2002). Utility of the Mayo End-Stage Liver Disease (Meld) Score in Assessing Prognosis of Patients with Alcoholic Hepatitis. BMC Gastroenterol.

[15] Luca A, Angermayr B, Bertolini G, Koenig F, Vizzini G, Ploner M (2007). An Integrated Meld Model Including Serum Sodium and Age Improves the Prediction of Early Mortality in Patients with Cirrhosis. Liver Transpl.

[16] Wang J, Wang AJ, Li BM, Liu ZJ, Chen L, Wang H (2014). Meld-Na:Effective in Predicting Rebleeding in Cirrhosis after Cessation of Esophageal Variceal Hemorrhage by Endoscopic Therapy. J Clin Gastroenterol.

[17] Olivas P, Soler-Perromat A, Tellez L, Carrion JA, Alvarado-Tapias E, Ferrusquia-Acosta J (2024). Persistent Varices in Cured Patients:Understanding the Role of Hepatic Venous Pressure Gradient. JHEP Rep.

[18] Wang M, Shui AM, Huang CY, Kappus MR, Rahimi R, Verna EC (2024). The Liver Frailty Index Enhances Mortality Risk Prediction above and Beyond Meld 3.0 Alone. Liver Transpl.

[19] Ge J, Kim WR, Lai JC, Kwong AJ (2022). “Beyond Meld”- Emerging Strategies and Technologies for Improving Mortality Prediction, Organ Allocation and Outcomes in Liver Transplantation. J Hepatol.

[20] Majumdar A, Pinzani M (2016). The Holy Grail of a Biomarker for “Liver Function”. Clin Liver Dis (Hoboken).

[21] Chetwood JD, Wells MG, Tsoutsman T, Pulitano C, Crawford MD, Liu K (2022). Meld-Grail and Meld-Grail-Na Are Not Superior to Meld or Meld-Na in Predicting Liver Transplant Waiting List Mortality at a Single-Center Level. Transplant Direct.

[22] Asrani SK, Jennings LW, Kim WR, Kamath PS, Levitsky J, Nadim MK (2020). Meld-Grail-Na:Glomerular Filtration Rate and Mortality on Liver-Transplant Waiting List. Hepatology.

